# Forecasting individual breast cancer risk using plasma metabolomics and biocontours

**DOI:** 10.1007/s11306-015-0793-8

**Published:** 2015-03-10

**Authors:** Rasmus Bro, Maja H. Kamstrup-Nielsen, Søren Balling Engelsen, Francesco Savorani, Morten A. Rasmussen, Louise Hansen, Anja Olsen, Anne Tjønneland, Lars Ove Dragsted

**Affiliations:** 1 Department of Food Science, University of Copenhagen, Rolighedsvej 26, 1958 Frederiksberg, Denmark; 2 Danish Cancer Society Research Center, Strandboulevarden 49, 2100 Copenhagen, Denmark; 3 Department of Nutrition, Exercise and Sports, University of Copenhagen, Rolighedsvej 26, 1958 Frederiksberg, Denmark

**Keywords:** Metabolomics, Early detection, Multivariate analysis, Plasma, Danish diet, Cancer and health cohort, Chemometrics, NMR

## Abstract

Breast cancer is a major cause of death for women. To improve treatment, current oncology research focuses on discovering and validating new biomarkers for early detection of cancer; so far with limited success. Metabolic profiling of plasma samples and auxiliary lifestyle information was combined by chemometric data fusion. It was possible to create a *biocontour*, which we define as a complex pattern of relevant biological and phenotypic information. While single markers or known risk factors have close to no predictive value, the developed biocontour provides a *forecast* which, *several years before diagnosis,* is on par with how well most current biomarkers can diagnose *current* cancer. Hence, while e.g. mammography can diagnose current cancer with a sensitivity and specificity of around 75 %, the currently developed biocontour can predict that there is an increased risk that breast cancer will develop in a subject 2–5 years after the sample is taken with sensitivity and specificity well above 80 %. The model was built on data obtained in 1993–1996 and tested on persons sampled a year later in 1997. Metabolic forecasting of cancer by biocontours opens new possibilities for early prediction of individual cancer risk and thus for efficient screening. This may provide new avenues for research into disease mechanisms.

**Electronic supplementary material:**

The online version of this article (doi:10.1007/s11306-015-0793-8) contains supplementary material, which is available to authorized users.

## Introduction

Breast cancer is the major cause of death for women in the first decade after menopause. Despite insight into several disease risk factors, these explain only a minor fraction of the incident cases. Continuous improvements in sensitivity, resolution and precision of modern explorative technologies like metabolomics continuously increase the potential to identify additional risk factors. More importantly, the platforms also form a basis for prediction modeling at the individual level, i.e. individual prediction of disease risk. This translational aspect has not been exploited to any large extent until now, primarily due to the inherent difficulties associated with the technologies. Omics-based biomarker profiling is a complex and truly multi-disciplinary subject.

Proliferation of the tumor at time of diagnosis is probably the factor with the greatest effect on survival rates amongst cancer patients. Consequently, an important focus in cancer research is to improve the ability to detect malignancy prior to the stage where the tumor has evolved into a clinically detectable disease. Breast cancer is the most common type of cancer diagnosed among women in the Western part of the world. In Europe, 458,337 women were diagnosed with breast cancer in 2012 and 131,259 women died of breast cancer (Ferlay et al. 2012). Worldwide, close to 1.4 million women are diagnosed with breast cancer each year and approximately one-third die from this disease. To facilitate detection of breast cancer prior to the occurrence of clinical symptoms, many Western countries have introduced mammography screening programs that are broadly aimed at middle-aged women. Mammography offers a fast diagnostic test for potential early stage malignancies. The risk of too many false positives in mammography screening, that is, detection of tumors that never progress to a stage that will affect the wellbeing of the patient has, however, been heavily discussed (Independent UK Panel on Breast Cancer Screening 2013). In the current analysis the cancer is not present (let alone diagnosed) at the time of the sampling but is diagnosed years later. It is the prediction of this later diagnosis of cancer that is the aim of this study. Such a method of early prediction of breast cancer risk at a time before diagnosis will have further substantial ethical implications but may also offer new leads for understanding cancer causation and for early detection of cancer.

## Methods

### Cancer cohort and samples

In the current project, samples from 838 Danish women enrolled in the Danish Diet, Cancer and Health (DCH) cohort have been analyzed. The cohort was established in the years 1993–1997 and consists of a total of 57,053 men and women free of cancer at the time of recruitment (Tjønneland et al. 2007). The DCH cohort is part of the European Prospective Investigation into Cancer and nutrition (EPIC) study including cohort participants from ten European countries. In the part of the cohort investigated here, half the women were diagnosed with breast cancer between time of enrolment and the chosen follow-up date (December 31, 2000). From the same cohort, an equal number of randomly selected women free of cancer during the same timespan, were selected as controls. These were not matched on age. Plasma samples were withdrawn in a non-fasting state, and citrate was used as anticoagulant. The samples were stored at −150/−80 degrees until analysis.

### Data collection and analysis—NMR

The plasma samples were analyzed by proton Nuclear Magnetic Resonance (^1^H NMR), see Online Supplemental Materials. The ^1^H NMR analytical platform (Beckonert et al. 2007) has several advantages compared to other common metabolomic analytical platforms. In particular, it is inherently quantitative and provides an unbiased and highly reproducible simultaneous observation of multiple metabolites. The so-called ‘curse of dimensionality’ (Bellman 1957) poses a practical hindrance for how much information can be obtained when few samples and many variables are measured. In the ^1^H NMR data, there are resonances from each hydrogen atom in hundreds of molecules sampled in several thousand variables. The high number of variables increases the risk of *spurious correlations* and this is a fundamental problem in non-targeted and comprehensive analyses (Kjeldahl and Bro 2010). A way to counter the influence of spurious correlations, is to have a sufficient number of samples compared to the number of variables and to avoid inflating the number of variables if possible. In this case, the NMR spectra were transformed into a less redundant representation by using integrals of 189 identified spectral regions. These regions were further reduced to 129 variables as some regions contained resonances from the same chemical compounds (see Online Supplementary Information). Each individual region was carefully selected and assessed and, in order to avoid selection bias, the best approach for integrating was decided for each in a blinded way, i.e. without any knowledge of the outcome (cancer status).

### Data collection and analysis—additional variables

In addition to the NMR data, 47 variables contained information about the lifestyle and phenotype of the subjects, resulting in a total of 176 variables. A complete list of these additional parameters, which mainly relate to anthropometrics, life style habits such as smoking, alcohol intake and dietary habits, can be found in the Supplementary Information. These variables will be named ‘lifestyle’ variables subsequently for convenience although it is to be remembered that the variables cover broader than just lifestyle.

### Model construction and validation

All models were built using the chemometric classification model, Partial Least Squares Discriminant Analysis (PLS-DA) (Geladi and Kowalski 1986; Næs and Indahl 1998), only using samples taken in the period 1993–1996; 709 samples in total. Upon establishing the actual classification model on samples from this period, the model was applied to data from 129 samples subsequently obtained in 1997.

## Results and discussion—forecasting breast cancer status at follow-up

### Hormone replacement therapy

Consider the number of years using hormone replacement therapy (variable “HRT—years of use”). This is an established risk factor for breast cancer (Tjønneland et al. 2004). A linear discriminant analysis using just hormone replacement therapy yields a classification error of 42 % which is close to a random assignment. This shows the limited discriminatory power of this single risk marker. The present dataset is rather high in the number of samples and therefore also in statistical power. Null hypothesis testing of “HRT—years of use” reveals apparent strong results (p_HRT—years of use_ = 0.00001). Although this suggests real differences between cases and controls on the population level, it is clear from the actual classification that this variable offers *no* power in terms of predicting the future status (cancer/no cancer) of an individual.

### Biological pattern analysis

Rather than using single variables, it is imperative to use a sufficient number of relevant variables to reflect the *biological patterns* that relate to the given endpoint. The chemometric classification model PLS-DA allows building multivariate classification models with correlated variables. When combining all the 47 available lifestyle variables including “HRT—years of use”, a multivariate classification model can be obtained with a classification error of 40 %. This is not better than a model with only years of hormone treatment. None of these models have any real predictive power.

**Fig. 1:**
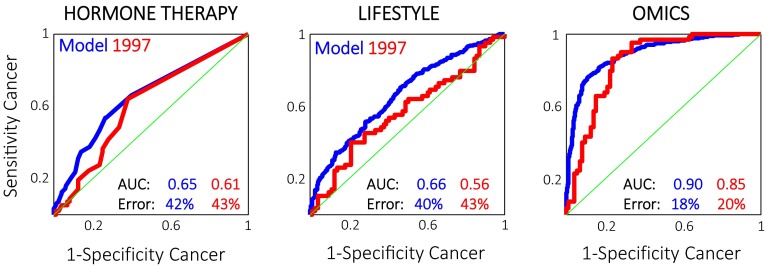
Resulting ROC curves from a univariate model (*left*), a model of the 47 lifestyle variables (*middle*) and the model based on all relevant data (*right*)

Instead of relying only on the traditional life style and risk markers, it is possible to do data fusion of the NMR and additional variables (Bro et al. 2013). By using variable selection based on forward selection (Andersen and Bro 2010; Nørgaard et al. 2000; Ståhle and Wold 1987) a subset of variables were selected one by one until the cross-validated prediction error did not improve (nine randomly selected segments averaged over seven repetitions). This led to a classification model using a total of 27 of the original variables. The resulting model provides a hitherto unseen effective means for forecasting breast cancer with an error of 18 %. The quality of all three mentioned models is given in Table 1 and associated receiver operator characteristics (ROC) curves (Zweig and Campbell 1993) in Fig. 1. A model based only on NMR was also evaluated and led to a model with a classification error of 22 %. Hence, the NMR part of the data by far contains the most important part of the information. In the obtained classification models, it was investigated if any *one* variable was crucial for the classification, but this was not the case. Instead, it is the pattern of biological data—a biocontour—which is required for accurately predicting the risk. In fact, any of the variables may be substituted without major loss of predictive power indicating substantial informational redundancy in the data set.

**Table 1:** Classification results using a single risk factor (years of hormone treatment), a palette of lifestyle variables (47 in total) and using NMR data together with additional data

	AUC	Classification error (%)	
		Calibration	1997 Samples
Hormone replacement therapy	0.65	42	43
47 Risk factors and phenotypes	0.68	40	43
All data	0.89	18	20

*AUC* area under the curve (where one indicates a perfect classification and 0.5 indicates no predictive power)

### Model validation

Rigorous validation is of utmost importance, especially when the variable to sample ratio is high and the relevant signals are deeply buried in the data. Two approaches are often used for validating biological models. One is interpretation of the model which may also give clues to more fundamental insight on cancer while another approach for validation is to test the model on new data. Both are pursued here.

**Fig. 2:**
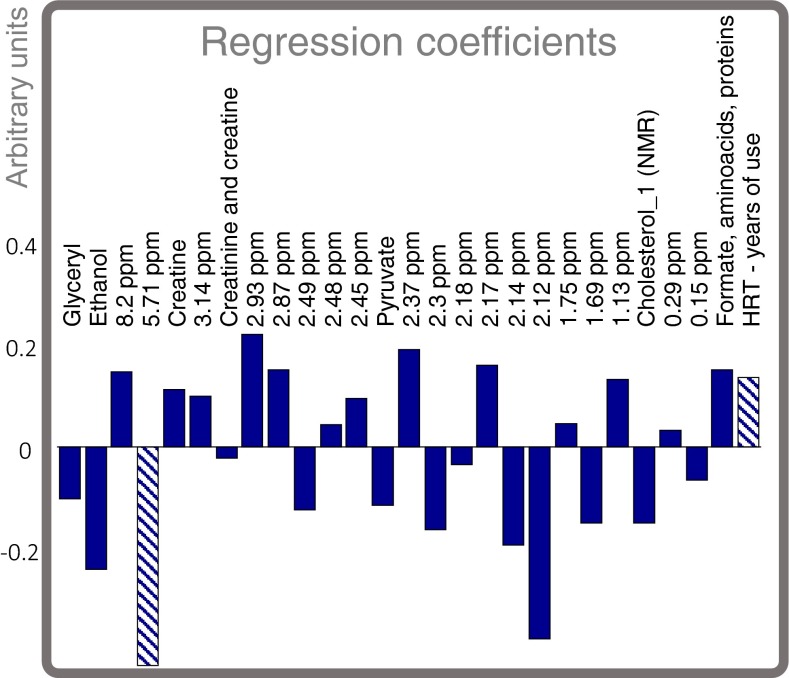
Regression coefficients of the classification model. Two variables are *highlighted* as they are discussed below

So-called score plots can be deceiving as the extent of overfitting is difficult to assess (Kjeldahl and Bro 2010). The classification model is based on a PLS-DA model of 27 selected variables. The regression vector indicates the importance of the 27 variables as shown in Fig. 2 and may serve as a basis for understanding the model.

For example, it is observed that hormone replacement therapy (HRT—years of use) has a large positive regression coefficient confirming the fact that hormone treatment is considered a risk factor. Due to spectral overlap it is difficult to uniquely identify specific NMR variables, but one interesting case is constituted by a peak at 5.71 ppm. It seems that it is negatively associated with cancer incidence.

Further investigations aimed at assigning the NMR signal, led to the conclusion that the chemical shift fits that of cis-aconitate which is an intermediate molecule in the Krebs cycle (tricarboxilic-acid cycle) in between the isomerization of citrate to isocitrate and also known to be relevant in relation to cancer (Wallace 2012).

Note that Ethanol (measured by NMR) has a negative regression coefficient. Ethanol intake in general, has a known positive effect on development of breast cancer, so the negative coefficient may at first be disturbing. However, the regression coefficients in an empirically based regression model can be opposite (or absent) to the known causal direction. This is also known as Simpson’s paradox and it is a very common, quite simple and yet often overlooked phenomenon (Simpson 1951). Regression coefficients must be interpreted with much more care when there is no experimental design behind the data. In fact several cautionary warnings are appropriate when dealing with interpretation of untargeted empirical models:

- Single variables with high coefficients (or correlations) are not necessarily risk factors in their own rights. For example, according to the current data, HRT may also be seen as preventive for some persons in terms of cancer depending on other variables. The results in Table 1 clearly show that hormone treatment in itself implies very little individual risk as judged from these data.
- The variables included in the model above are not to be expected as the most likely candidates for explaining *why* we can predict cancer. The variables are merely accurate indicators of the biocontour and nothing statistically or biologically suggests that these would offer the most appropriate explanation. For example HRT (or any other variable) were found to be replaceable by other variables yet still maintaining the same classification power. We call this the *cage of covariance*; maintenance of homeostasis as a result of a complex metabolic network implies that many factors may be equally influenced by any biochemical change. This is the downstream consequence of pleiotropy.

### The concept of a biocontour

The main virtue of NMR is that it measures a fair number of high concentration (bulk) plasma metabolites with high precision and reproducibility. Apparently the bulk plasma metabolome is perturbed in the subjects which later develop breast cancer and NMR measures enough metabolites with high precision that the biological cage of covariance can reflect the perturbed plasma metabolome, i.e. an altered biological contour of the variables at large.

Theories may well be developed based on the totality of the biocontour—not just the selected 27 variables—once its variables have been unequivocally identified to allow bioinformatics analyses. It would be possible that several factors could point to metabolic pathways important for breast cancer prediction and could therefore form an important avenue for novel mechanistic research into a potential relation with causation.

While identifying the hundreds of metabolites in the current biocontour can be interesting, it will also be a time-consuming endeavor. We suggest that the biocontour has immediate importance in its own right as a predictor for future cancer regardless of the level to which the contour can provide mechanistic understanding. Hence, as a more powerful means for validating the model, the real classification power is assessed using independent samples. In Table 1, it is shown that when the model based on NMR and lifestyle data is used on samples from a subsequent year, the predictive power is maintained. This provides very strong evidence for the robustness and validity of the current predictive finding. The obvious next step will be to investigate, if the model is indicative of cancer in general or just breast cancer. The data set also contains information on additional 428 colon cancer cases and we have analysed these samples as well, but there is no predictive power in terms of colon cancer in the data (results not shown). More investigations should be performed in other cohorts for further validating the specificity of the current breast cancer prediction model.

## Conclusion

We have described a biocontour that can forecast individual diagnosis of breast cancer several years ahead. It has been subjected to strong validation. Its applicability for other populations of women with other diets, lifestyles, medications and habits is unknown and should be investigated before attempting to translate our model into clinical use. The perspectives in early detection of other cancers and chronic diseases by use of biocontours from human samples fused with life style variables from apparently healthy persons are of worldwide importance. We advocate that biocontours get a much more prominent role in disease diagnostics, including cancer prediction and as potential new leads for complex biological interactions in disease causation.

## Electronic supplementary material

Below is the link to the electronic supplementary material.

Supplementary material 1 (DOCX 69 kb)
