# Supplementary material for: Forecasting individual breast cancer risk using plasma metabolomics and biocontours
Source: Metabolomics. 2015 Mar 10;11(5):1376–80. doi: 10.1007/s11306-015-0793-8 (PMC4559100; doi:10.1007/s11306-015-0793-8)
Supplement: Supplementary file 1 — Supplementary material 1 (DOCX 69 kb) [file 11306_2015_793_MOESM1_ESM.docx]

Supplementary Information

*Study population*
The present study is based on data from the prospective Danish Diet, Cancer and Health (DCH) cohort study. A total of 57,053 men and women were enrolled into the cohort in 1993-1997 and were included if they fulfilled the following criteria: age between 50-64 years, born in Denmark and no previous cancer diagnosis in the Danish Cancer Registry. A detailed food frequency questionnaire (FFQ) and lifestyle questionnaire were filled in by each participant. Development and validation of the FFQ is described elsewhere. The FFQ contained questions regarding 192 food and beverage items and was developed to obtain information on the participants’ habitual diet during the preceding year. Biological and anthropometric measurements were taken including height (m) and weight (kg), from which body mass index (BMI) was calculated (kg/m^2^) as well as a non-fasting 30 mL blood sample. Citrate was used as the anticoagulant. The blood samples were spun and divided into fractions of citrate plasma, serum, red blood cells, and buffy coat and stored in 1 mL tubes. All samples were processed and frozen within two hours at -20°C and were ultimately transferred to liquid nitrogen vapor (max. -150°C). Shortly before NMR analysis, samples were retrieved from the bio-bank and stored at -80°C. For the current project, the plasma fraction was used. A thorough description of data collection has been published elsewhere (Tjønneland, A. et al. 2007).

From the DCH cohort, a combined study population of 3510 persons was created, consisting of three case groups: breast cancer (433), colon cancer (428) and coronary heart disease (1149) as well as a sub-cohort (1500 persons) to be used as a reference group for all. After exclusions due to insufficient sample volume or low data quality, the total study population consisted of 3,419 persons, of which 419 were breast cancer cases. As only the breast cancer case group was used for this project, no further mention will be made of the two remaining case groups. The sub-cohort consists of 1500 persons (750 men, 750 women) who were chosen randomly in the DCH cohort, and only the women in the sub-cohort were used in this investigation.

*Follow-up and ascertainment of cases*

Information on breast cancer incidence was obtained by linkage of the personal identification number of each participant to the Danish Cancer Registry, and follow-up was nearly complete (99.8%). All cohort members were followed up for primary breast cancer diagnosis from the date of their visit to the study clinic until the date of diagnosis of any cancer (except for non-melanoma skin cancer), date of death or emigration, or December 31, 2000, whichever came first. Furthermore, the Danish Breast Cancer Co-operative Group (DBCG) has information on estrogen receptor (ER) status (ER+ or ER-) for approximately 90% of all Danish breast cancer cases, and ER status was available for 384 of the 419 breast cancer cases in this study.

*NMR analysis*

Plasma samples were thawed on ice and 300 µL plasma were mixed with 300 µL phosphate buffer (pH 7.4) containing trimethylsilyl propionate (TSP) as reference signal and D2O for the lock signal and then transferred to 5 mm NMR tubes. NMR analysis was performed with a Bruker Avance III 600 spectrometer (Bruker Biospin Gmbh, Rheinstetten, Germany) operating at a Larmor frequency of 600.13 MHz for protons, equipped with a double tuned cryo-probe (TCI) set for 5 mm sample tubes and a cooled autosampler (SampleJet). Spectra were acquired from all plasma samples using both the Carr-Purcell Meiboom-Gill (CPMG) experiment and the NOESY-presat one according to Beckonert et al. (Beckonert, O. et al. 2007). All experiments were performed at 310K with a fixed receiver gain (RG), which was determined through initial tests. Each free induction decay (FID) was collected using a total of 128 scans. Prior to Fourier transformation the FIS’s were zerofilled to 64 K points and apodized by 0.3 Hz Lorentzian line broadening and thereafter baseline- and phase corrected automatically. To correct for spectral misalignment the entire dataset was globally aligned with respect to the α-D-glucose signal around 5.25 ppm using the icoshift algorithm (Savorani, F. et al. 2010). The plasma samples were prepared and measured using a standardized and highly automated procedure on the basis of the guidelines introduced by Bechonert et al (Beckonert, O. et al. 2007). In the NOESY-presat data, the water-resonance is suppressed, but otherwise all components are observed in a quantitative manner. In the CPMG-presat data, also resonances from large molecules such as proteins are suppressed, which facilitates easier identification of small molecules.

The NMR spectra have been subjectively evaluated by spectroscopists and data analysts in order to exclude as many noise regions from the data as possible and to include all peaks in the most parsimonious manner. The spectroscopists and data analysts were blinded to the case/control status. Many of the peaks were present in both the CPMG-presat and NOESY-presat spectra and were only selected in one of these. The majority of the peaks were selected from CPMG-presat as there was generally a better defined baseline in these spectra due to the suppression of protein resonances. In total 181 intervals from CPMG-presat and eight intervals from NOESY-presat were selected. The 189 intervals were individually baseline corrected and integrated by means of either Multivariate Curve Resolution (MCR) (Xie, Y. L. et al. 1998), the area under the peak or the height of the peak. Peak height provides a reasonable estimate of concentration (up to a scaling) when the line shapes of the peak in different samples are similar. Peak height has been used for integrating when e.g. baseline resolution was difficult to achieve. Integrating peaks leads to one value instead of a lineshape for each peak. For one peak, two MCR components were used instead of one. Many intervals represent the same compounds. For the intervals which have been assigned (Nicholson, J. K. et al. 1995), those originating from the same molecule have been summed, meaning that all assigned compounds are only represented once. Consequently, the NMR data contribute with a total of 129 discrete integrated variables. The assigned peaks and areas in the NMR spectrum can be seen in Figure 1.

|  |
| --- |
|  |
|  |

Figure 1. ^1^H CPMG-presat NMR spectra with annotation for selected spectral regions: ^a)^ 5.6-9.2 ppm, ^b)^ 2.7-5.6 ppm, and ^c)^ 0.3-2.7 ppm. In each region, the sub-spectrum is vertically scaled according to the most intense resonance. The average spectrum is shown.

*Additional data*

The additional variables included in the data analysis stem from the dietary and lifestyle questionnaires that were collected at baseline in the DCH cohort and from clinical markers obtained initially. More than 1000 variables were potentially available; however, of these variables, some are derived directly from the questionnaire, while others are summed or cumulated variables. We chose the ones that were deemed most relevant through a careful selection process prior to the data analysis. A total of 47 dietary and lifestyle variables were chosen based on knowledge of factors known or speculated to be important for cancer or heart disease development. The additional data included in the data analysis contribute with additional information regarding the life styles of the persons and are shown below with a short description (Table 1).

*Table 1. Additional variables included in the classification model.*

| Variable | **Explanation** |
| --- | --- |
| Age >35 at first birth or no births | Older than 35 when having the first child OR have not given birth at all |
| Age at birth of first child | Age at first child birth |
| Alcohol intake | Alcohol (grams/day) |
| Alcohol intake (cumulated) | Cumulative alcohol, excluding pauses (units/week) |
| Amount of body fat | Fat weight (amount of fat mass in the body) (kg) |
| BMI | Body mass index (kg/m^2^) |
| Carbohydrate intake | Carbohydrates (grams/day) |
| Coffee intake | Coffee (grams/day) |
| Dietary fibre intake | Dietary fibre (grams/day) |
| Energy intake | Total energy intake, incl. alcohol (kJ/day) |
| Fat intake | Fat (grams/day) |
| Fat pct. | Fat percent in diet |
| Fatty dairy products | Dairy products, fatty (grams/day) |
| Fruit intake (no juices) | Fruits (excl. juices) (grams/day) |
| Fruits (incl. juice) | All fruits (incl. juices) (grams/day) |
| Glycemic index (carbohydrates) | Overall glycemic index (based on all carbohydrates) (grams/day) |
| Glycemic load (carbohydrates) | Overall glycemic load (based on all carbohydrates) (grams/day) |
| Height | Standing height (cm) |
| High level school | Highest level school education |
| Hip circumference | Hip circumference (cm) |
| HRT – years of use | Years of HRT use |
| Intake of rye bread | Rye bread (grams/day) |
| Juice intake (fruit and vegs) | Juices (fruit and vegetable) (grams/day) |
| Lean dairy products | Dairy products, lean (grams/day) |
| Level of serum cholesterol | Level of serum Cholesterol (mmol/L) |
| Low level school | Low level school education |
| Marine omega-3 | Marine fats (n-3) in diet (grams/day) |
| Medium level school | Medium level school education |
| Mono unsaturated fatty acids | Monounsaturated fat (grams/day) |
| Number of births | Number of births |
| Poly unsaturated fatty acids | Polyunsaturated fat (grams/day) |
| Potato intake | All potatoes (grams/day) |
| Processed meat | Processed meat (grams/day) |
| Protein intake | Protein (grams/day) |
| Red meat | Red meat (grams/day) |
| Saturated fatty acids | Saturated fat (grams/day) |
| Sugar intake | Total, all sugars (grams/day) |
| Syst. Blood pressure | Blood pressure, systolic |
| Total exercise (MET score) | MET-score, hours/week |
| User of nsaid | Current user of NSAIDS, including aspirin (self-reported) |
| Vegetables (incl. juice) | All vegetables (incl. juices) (grams/day) |
| Water intake | Water (grams/day) |
| Waist circumference | Waist circumference (cm) |
| Waist/hip ratio | Ratio of waist to hip |
| Weight | Weight (kg) |
| Years of quitting smoking | Time since smoking cessation (years) |
| Years of smoking | Smoking duration (years) |

*Data analysis*

All data preprocessing and data analysis was carried out in MATLAB 2013A, The MathWorks Inc., Natick, MA, 2013 and with the chemometric toolbox PLS_Toolbox 7.0.3, Eigenvector Research, Inc., Wenatchee, WA, 2013. After integration of peaks, the 129 peak variables and the 47 additional variables were kept in a single dataset. Before data analysis, the data was split into a calibration set of 709 samples from the period 1993-1996 and a test set of all 129 samples taken in the subsequent year 1997.

Variables for the calibration model were selected based on interval Partial Least Squares regression – iPLS (Nørgaard, L. et al. 2000) using only the calibration data and scaling each variable to unit standard deviation within the cross-validation. Variables were added one by one in a forward selection as long as the model improved. As a criterion for selection, the cross-validated classification error was used in an automated fashion. Cross-validation was performed using nine randomly selected segments and this cross-validation was repeated seven times for each step. This quite conservative repetition of randomly selected segments provides a practical means for avoiding spurious correlations but has no effect on luring or indirect correlations.

Upon selecting variables, a final PLS discriminant model was made on the selected variables using autoscaling and selecting the number of PLS components based on the same cross-validation as above and on minimum cross-validated classification error. This model was used for predicting the class assignments on the test set with the prior selected variables.
